# Effect of UV-C Radiation, Ultra-Sonication Electromagnetic Field and Microwaves on Changes in Polyphenolic Compounds in Chokeberry (*Aronia melanocarpa*)

**DOI:** 10.3390/molecules22071161

**Published:** 2017-07-12

**Authors:** Tomasz Cebulak, Jan Oszmiański, Ireneusz Kapusta, Sabina Lachowicz

**Affiliations:** 1 Department of Food Technology and Human Nutrition, Faculty of Biology and Agriculture, Rzeszów University, 4 Zelwerowicza Street, 35-601 Rzeszów, Poland; tomcebulak@gmail.com (T.C.); ikapusta@ur.edu.pl (I.K.); 2 Department of Fruit, Vegetable and Plant Nutraceutical Technology, Wrocław University of Environmental and Life Science, 37, Chełmońskiego Street, 51-630 Wrocław, Poland; jan.oszmianski@upwr.edu.pl

**Keywords:** chokeberry, abiotic stress, UV-C radiation, electromagnetic field, microwave radiation, ultrasound, UPLC-PDA-MS/MS, polyphenolic compounds, antioxidant activity

## Abstract

Chokeberry fruits are highly valued for their high content of polyphenolic compounds. The use of such abiotic stress factors as UV-C radiation, an electromagnetic field, microwave radiation, and ultrasound, at different operation times, caused differentiation in the contents of anthocyanins, phenolic acids, flavonols, and flavan-3-ols. Samples were analyzed for contents of polyphenolics with ultra-performance liquid chromatography and photodiode detector-quadrupole/time-of-flight mass spectrometry (UPLC-PDA-MS/MS). The analysis showed that after exposure to abiotic stress factors, the concentration of anthocyanins ranged from 3587 to 6316 mg/100 g dry matter (dm) that constituted, on average, 67.6% of all identified polyphenolic compounds. The second investigated group included phenolic acids with the contents ranging between 1480 and 2444 mg/100 g dm (26.5%); then flavonols within the range of 133 to 243 mg/100 g dm (3.7%), and finally flavan-3-ols fluctuated between 191 and 369 mg/100 g dm (2.2%). The use of abiotic stress factors such as UV-C radiation, microwaves and ultrasound field, in most cases contributed to an increase in the content of the particular polyphenolic compounds in black chokeberry. Under the influence of these factors, increases were observed: in anthocyanin content, of 22%; in phenolic acids, of 20%; in flavonols, of 43%; and in flavan-3-ols, of 30%. Only the use of the electromagnetic field caused a decrease in the content of the examined polyphenolic compounds.

## 1. Introduction

Black chokeberry (*Aronia melanocarpa*) (Michx. Elliott), a shrub belonging to the *Rosaceae* family, derives from the eastern coast of the North American continent, where it grows wild. In Central Europe black chokeberry is cultivated on plantations and occasionally is planted in forests. The shrub reaches a height from 0.5 up to 3 m. Its ripe fruits are used to produce jams, juices, wine as well as for the production of anthocyanins. They owe their biological quality to a high level of minerals, mainly potassium (2.9 g/kg), and to the contents of sugar, including glucose (47.1 g/kg), fructose (37.8 g/kg), and sorbitol (66.1 g/kg) [1]. Chokeberries are also abundant in B-group vitamins and polyphenols [2]. The content of phenolic compounds in the chokeberry fruit fluctuate between 40 and 70 mg/g dm, of which 50% are anthocyanins. Oszmiański and Wojdyło [3] reports that cyanidin-3-galactoside, cyanidin 3-arabinoside, cyanidin 3-glucoside and cyanidin 3-xyloside are major polyphenolic compounds. According to the literature [4,5], chokeberries have health-promoting effects on a number of metabolic and immune diseases, particularly those related to oxidative stress, including cardiovascular and gastrointestinal diseases, cancer, lipid disorders and antiviral activity. Studies of Ho et al. [6] have shown that regular consumption of fresh and processed chokeberry fruits prevents cardiovascular disease, regulates blood pressure, reduces the side effects of drugs used in anticancer treatment, alleviates radiotherapy effects and immunizes the skin against harmful UV radiation. Fang et al. [7] claims that in the case of chokeberry such an effect is mainly related to a high content of polyphenolic compounds, particularly anthocyanins.

An increase in nutritional awareness makes the consumer’s choice more attentive and is also a reason to search for food with high biological value. Hence, particular attention is drawn to the products characterized by high antioxidant activity due to a scavenging effect of their compounds on the reactive oxygen and nitrogen, which occurs in the body due to the metabolic reactions as well as in the case of inflammation and neutralization of xenobiotics [8]. Among the compounds exhibiting the strongest antioxidant activity, there are primarily polyphenols [9,10]. Plants metabolize phenolic compounds from the precursors of phenylalanine and shikimic acid [11,12]. Their synthesis is especially intensified during biotic and abiotic stress [13]. As was reported by Ke and Saltveit [14], the action of ethylene and pathogens has the effect of an increase in polyphenols in iceberg lettuce [14]. The later findings of Hasanuzzaman et al. [15] and Yu et al. [16], who examined mainly vegetables, indicate that the heat stress (via elevating the temperature of plant tissue) and UV irradiation lead to changes in polyphenol content, the range of which depends on power and, sometimes, the operating time of abiotic stress factors [15,16].

The aim of the present study was to determine the effect of abiotic stress factors such as UV-C radiation, electromagnetic field, microwave field and ultrasounds on changes in the levels of polyphenolic compounds in fresh chokeberry fruits.

## 2. Results

**Figure 1:**
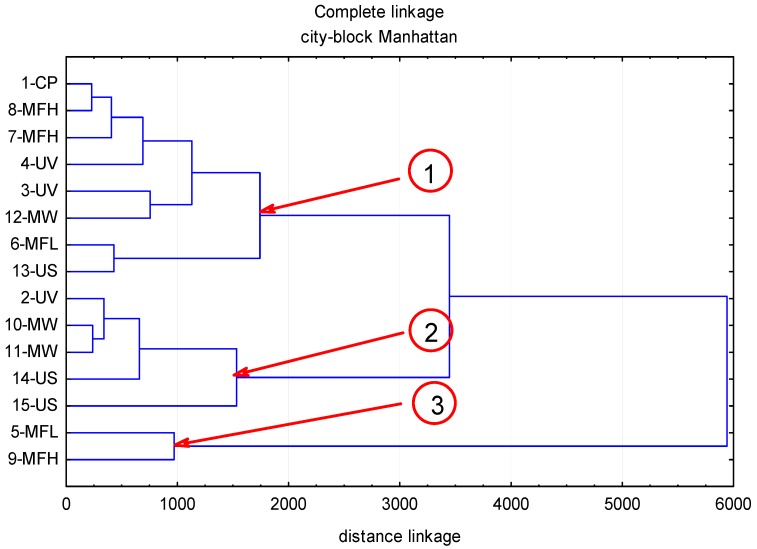
The influence of abiotic stress factors (UV-C electromagnetic field of the microwave field, ultrasound) on the content of anthocyanins in chokeberry fruits.

**Figure 2:**
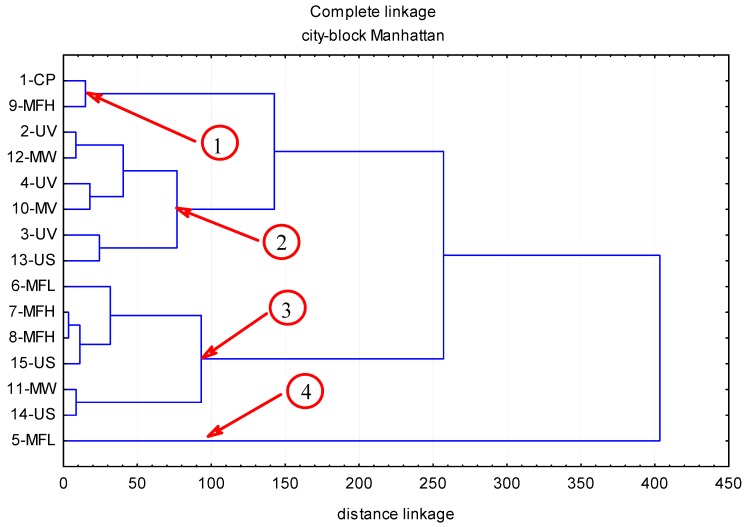
The influence of abiotic stress factors (UV-C electromagnetic field of the microwave field, ultrasound) on the content of chlorogenic acid in chokeberry fruits.

**Figure 3:**
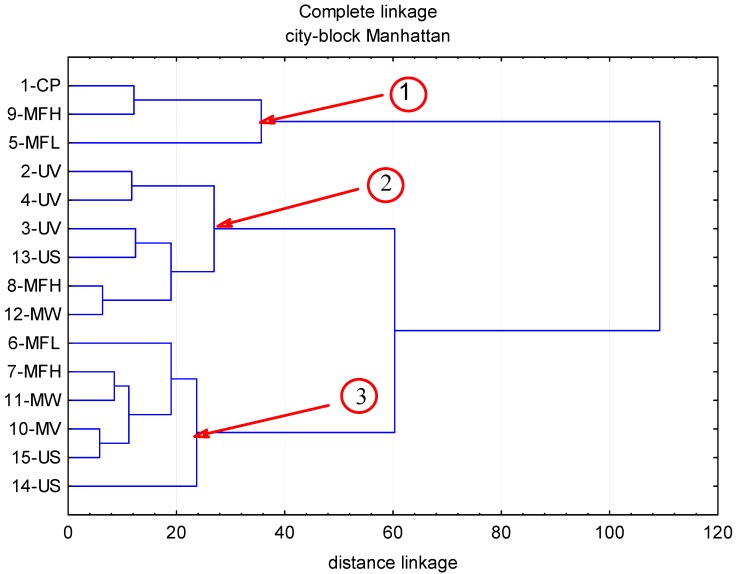
The influence of abiotic stress factors (UV-C electromagnetic field of the microwave field, ultrasound) on the content of flavonols in chokeberry fruits.

**Figure 4:**
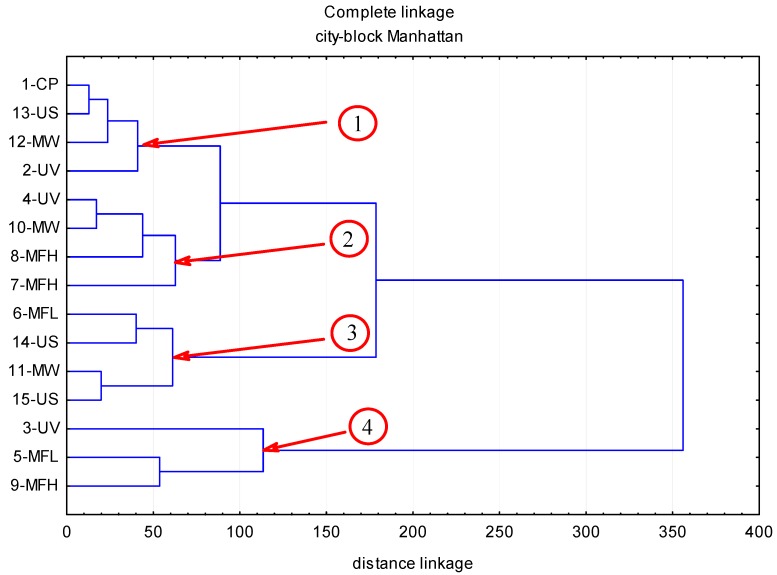
The influence of abiotic stress factors (UV-C electromagnetic field of the microwave field, ultrasound) on the content of flavan-3-ols in chokeberry fruits.

The contents of polyphenolic compounds in the chokeberry fruit subjected to abiotic stress factors are given in Table 1, while Figure 1, Figure 2, Figure 3 and Figure 4 illustrate cluster analysis by showing clusters of the abiotic stress factors. The ultra-performance liquid chromatography time-of-flight mass spectrometry (UPLC-PDA-MS/MS) analysis demonstrated that anthocyanins were a predominant group of polyphenolic compounds in chokeberry fruit; their content ranged from 3587 to 6316 mg/100 g dm and this was, on average, 67.6% of all identified polyphenolic compounds. Then were phenolic acids (from 1480 to 2444 mg/100 g dm; 26.5%), and flavonols (from 133 to 243 mg/100 g dm; 3.7%). The fourth group comprised flavan-3-ols (from 191 to 369 mg/100 g dm; 2.2%).

### 2.1. Anthocyanins

In fresh chokeberry fruits and those treated with abiotic factors, eight anthocyanin compounds have been identified (Table 1 and Table 2), of which two—cyanidin-3-*O*-galctoside and cyanidin-3-*O*-arabinoside—comprised 93% of total anthocyanin content, which in the examined samples ranged from 3587 mg/100 g dm (5-MFL) to 6316 mg/100 g dm (15-US). This corresponds to the percentage diversity from −31% to +22%, compared to the control sample (CP-1). Of the anthocyanins determined, the largest loss (54%) was found for cyanidin-3-pentoside-(epi)catechin after 5 min treatment with the electromagnetic field of 40 mT and 50 Hz (9 MFH). The highest increase (of 233%) was recorded for cyanidin after 8 min application of ultrasounds of the power of 800 W and the frequency of 40 Hz (15 US). The use of cluster analysis allowed the identification of three areas of mutual relationships between abiotic stress factors and the level of anthocyanins in the fruits of chokeberry (Figure 1). In the first area, grouped around the control sample, there were the factors exhibiting small positive effects of abiotic stress on the level of anthocyanins in black chokeberry. This area, with the mean 12.5% increase in anthocyanin content, owed its shape to the following factors: MFH 8-7-MFH, 4-UV, UV-3, 12-MW, MFL-6 and 13-US. The second area, in which the average increase in the concentration of anthocyanins was 30%, was determined by 2-UV, 10-MW, 1-MW, 14-US, and 1-US agents. The third area comprised of the factors 5-MFL and 9-MFH, which had a distinct adverse effect on the level of anthocyanins reducing their amount, on average, of 35.5%.

### 2.2. Phenolic Acids

Of the six identified phenolic acids with ultra-performance liquid chromatography and photodiode detector-quadrupole/time-of-flight mass spectrometry (UPLC-PDA-MS/MS), neochlorogenic acid and chlorogenic acid accounted for 98% of all the phenolic acids (Table 1 and Table 2). Abiotic stress factors had an effect on the differentiation of the concentration of phenolic acids in chokeberry fruit; in the samples not exposed to stress factors (control sample), the total content of phenolic acids was up to 2031 mg/100 g dm. In turn, when applying abiotic stress factors, their concentration varied in the range of 1480 mg/100 g dm (MFL5) to 2444 mg/100 g dm (14-US). Among the phenolic acids examined, di-caffeic quinic acid II was the most sensitive to the applied stress factors. In comparison with the control sample, changes in its concentrations fluctuated between −67% (UV-2) and +74% (15-US), which accounted for 0.28 and 1.5 mg/100 g when expressed in dm. Projection of an effect of abiotic stress factors onto the dendrogram’s plane (Figure 2) reveals inter-agent dependencies associated with the diversity in the contents of phenolic acids in chokeberry fruit. The structure of inter-group linkages in the cluster analysis resulting from the comparison of 15 different factors allowed 4 clusters to be found, which were linked by mutual relationships. Clusters 1 and 4 were associated with the factors lowering the concentration of phenolic acids in the examined fruit, while clusters 2 and 3 related to the factors increasing the level of these compounds. The first cluster’s space included the agent 9-MFH, which reduced the content of phenolic acids in chokeberry, on average, by 13%; whereas, due to the agent 5-MFL, representing cluster 4, their amount decreased, on average, by 27%. In the second cluster there are the factors: 2-UV, 12-MW, 4-UV, 10-MV, 3-UV, and 13-US, which contributed to a slight, on average, 2% increase in phenolic acids in the examined material. The space of the third cluster refers to the factors 6-MFL, 7-MFH, 11-MW, 14-US and 15-US, which markedly (on average by 16%) increased the level of phenolic acids in the examined material.

### 2.3. Flavonols

Total content of flavonols ranged from 133 (5-MFL) to 243 mg/100 g dm (14-US), as indicated in Table 1. In the control sample, the mean content of flavonols was 169 mg/100 g dry matter. Thus, stress agents caused changes in the level of these compounds in the range of −21% to +44%. This table also shows that 11 flavonols have been identified, of which, quercetin-3-*O*-galctoside dominated (23% of all the flavonols determined). It has been revealed that the 30 min exposure to a 150-μT electromagnetic field of a frequency of 100 Hz (5-MFL) contributed to the −21% reduction in the level of total flavonols compared to the control. In contrast, the 4 min ultrasound (US) treatment at 800 W and 40 Hz (14-US) led to a 44% increase in flavonols. The highest decline (−28%) was recorded in quercetin-3-robinobioside content, while the highest increase (+87%) was in isorhamnetin rhamnosyl-hexoside isomer. The plane of inter-agent dependences on the dendrogram (Figure 3) allowed the identification of three clusters illustrating inter-agent interactions. In the first one there were the agents closely related to the control sample as well as 9-MFH and 5-MFL; the mean level of flavonols determined therein increased by 8% compared to the control sample. Clusters 2 and 3 grouped the agents responsible for a substantial increase in the concentration of the compounds examined in the chokeberry fruit. Cluster 2 included the following agents: 2-UV, 4-UV, 3-UV, 13-US, 8-MFH, and 12-MW. Due to these agents, the level of flavonols in chokeberry fruit increased, on average, by 15%. The third space was formed by such agents as 6-MFL, 7-MFH, 11-MW, 10-MW, 15-US, and 14-US; in this case, an average 32% increase in flavonols was recorded compared to the control sample.

### 2.4. Flavan-3-ols

Analyses allowed the identification in black chokeberry of four flavan-3-ols and one dihydrochalcone (Table 1 and Table 2). Among the identified flavan-3-ols were: B-group procyanidin (procyanidin B2, (+)catechin, procyanidin B2, (−)epicatechin), dihydrochalkon eriodictyol 7-*O*-glucuronide. The control sample contained 284 mg the total examined compounds per 100 g of dm, while the chokeberry fruit exposed to stress agents were found to have between 191 and 369 mg/100 g dm (−33% to +30% compared to the control sample). Of the flavan-3-ols examined, B-group procyanidin having undergone treatment with the 5-MFL agent showed the largest loss (−71%) compared to the control sample: whereas, the highest increase (42%) was observed for (+)catechin exposed to an electromagnetic field (150 μT, 100 Hz) for 60 min (6-MFL). Cluster analysis allowed the separation of four clusters covering the factors of a similar impact on the levels of flavan-3-ols (Figure 4). Clusters 1 and 4 reflected the factors contributing to the reduction in flavan-3-ols content. On the other hand, data grouping expressed by clusters 2 and 3 show abiotic stress factors affecting a rise in the level of flavan-3-ols in black chokeberry. Cluster 1 included the factors 13-US, 12-MW and 2-UV, who caused a mean 1.5% drop in flavan-3-ol content compared to the control sample. In turn, aggregation of the 3-UV, 5-MFL and 9-MFH agents in cluster 4 revealed changes in the content of the analyzed compounds of −30%. The factors 4-UV, 10-MW, 8-MFH and 7-MFH have generated cluster 2 characterized by a mean 12% increase of flavan-3-ols; whereas, the factors 6-MFL, 14-US, 11-MW and 15-US formed a cluster 3, wherein an average increase in flavan-3-ols reached 24%.

## 3. Discussion

Plants, which are by their nature immobile, defend themselves against adverse environmental factors by developing complex mechanisms to protect their metabolism from the effects of biotic and abiotic stress. Plant defense strategies are mainly based on the modification of metabolic pathways leading to the production of the protective compounds against adverse effects of free radicals occurring as a result of stress factors [13,17]. In the case of plants, the increased production of enzymatic and non-enzymatic antioxidant substances is observed, among which are primarily phenolic compounds [18,19]. Scientific papers indicate that among polyphenols, flavonoids exhibit the strongest health-promoting properties [16,20]. Previous papers concern mainly plant response to stress during the growing season. According to Gill and Tuteja [13], mitochondria play a key role in cellular adaptation to reactive oxygen species (ROS). As a result of biotic and abiotic stress factors, the inner cellular temperature increases. This, in turn, induces an increase in the ROS production. In response, the plant produces intensively the compounds showing antioxidant potential. For example, Gill and Tuteja [13] found that numerous genes of the flavonoid biosynthesis are induced under stress conditions, particularly in the case of UV irradiation. In a view of the previously quoted works of Hasanuzzaman et al. [15], Yu et al. [16], and Gill and Tuteja [13], it should be expected that the use of the above-mentioned abiotic stress factors contributes to the temperature elevation in the plant tissue, thereby activating the antioxidant defense mechanisms. This, in turn, leads to an increased synthesis of polyphenolic compounds [13,15,16]. Thus, it seems reasonable to search for an answer to the question about the direction of changes occurring in polyphenol content in the chokeberry fruit exposed to abiotic stress factors. This work makes an attempt to determine the stimulating effect of the controlled application of the selected abiotic stress agents (UV-C radiation, electromagnetic waves, microwaves and ultrasound) on the content of polyphenolic compounds in chokeberry fruit. Of the abiotic stress factors, the effects of UV irradiation and ultra-sonication on the content of polyphenolic compounds in plants have been very broadly described in the literature. Chang-Hong et al. [21], who irradiated green tomatoes with various UV-C doses, found increases in the content of: gallic acid, by 21%; catechins, by 26%; chlorogenic acid, by 14%; caffeic acid, by 37%; quercetin, by 38%; and *p*-coumaric acid, by 36%, after the 35-day storage period. In the present study, changes in polyphenol content resulting from UV-C irradiation have also been noted. Their extent depended on the examined group of polyphenolic compounds and was: from −5% to +8%, for anthocyanins; from −4% to +7%, for the sum of phenolic acids; from +9% to +23%, for the sum of flavonols; and from −16% to +9%, for the sum of flavan-3-ols (Table 1 and Table 2). An above 30% increase was observed only in the case of cyanidin-3-hexoside-(epi)catechin (of 37%), while 20–30% increases were reported for such compounds as: quercetin-3-*O*-galactoside and cyanidin. As for phenolic acids, a change in chlorogenic acid content (of 12%) was found to be the largest. In the case of flavonols, the greatest increase (of 31%) was in quercetin-3-*O*-vicianoside content; whereas, with regard to flavan-3-ols, the highest (of 16%) was for B-group procyanidin. The largest changes towards an increase in the concentrations of the polyphenolic compounds were observed in the case of activity of ultrasounds and UV-C radiation. Smaller changes were linked with activity of MFL, MFH and MW. Only in the case of activity of MFH for the duration of 5 min and US in 2 min there were decreases in concentrations of polyphenolic compounds. The highest growth in the concentrations were observed among anthocyanins, in the case of compound **8**, at 8 min long activity of ultrasounds in the case of phenolic acids in compound **14**, and in the case of activity of ultrasounds for the duration of 8 min in flavonols, compound **23** at the activity of both ultrasounds for 4 min and microwave radiation for two min. The highest increase in concentrations of flavonols was found in compound **26** at 4 min activity of ultrasounds.

Ultrasounds produce the cavitation effect in cell organelles, resulting in an increase in cell membrane permeability via activation of calcium channels. This, in turn, enhances enzyme activity, probably responsible for the stimulation of the secondary metabolites’ synthesis [22]. Wu and Lin [23] revealed an increase in the level of polyphenolic compounds in vitro culture of the ginseng cell suspension due to 2 min exposure to ultrasound of a frequency of 385 Hz in a water bath, which was accompanied by increased activity of PPO and PO enzymes and lowered water content in the cells. This concurs with the findings of Santos et al. [24] who observed a 20% increase in total polyphenols after subjecting freshly cut slices of mango to ultrasonication (25 kHz, 30 min). In turn, Yu et al. [16], after 60 h storage of the romaine lettuce leaves treated with ultrasounds (25 kHz, 26 *W*/L) for 1, 2 and 3 min, noted increases in the levels of phenolic compounds of up to 22.5%, 16.3% and 17.9%, respectively. An increase in the content of polyphenolic compounds due to ultrasonication was also stated in the present study. Similar to the above-described influence of UV-C irradiation, ultrasounds also caused an increase in the content of polyphenolic compounds in chokeberry fruit. In most cases, an increase in polyphenolic compounds in the examined fruit was recorded. As for anthocyanins, their level was changing in dependence on the length of their exposure to US. The results obtained show that 8 min operation was optimal, as it caused an increase in the level of the majority of these compounds. An average increase in anthocyanin content was 22%; the highest, amounting to 44% (at 4 min ultrasonication), was recorded for cyanidin-3-hexoside-(epi) catechin. In a group of chlorogenic acids, changes ranged between −2% and 20%; the highest 32% increase was observed in the content of chlorogenic acid, after 4 min exposure to US. With regard to flavonols, their content after US exposure increased from 8 to 44%, compared to the control sample. In the case of flavan-3-ols, exposure to US led to the variation among the analyzed compounds of −2% to +30%. Randhir and Shetty [25] found that in germinating bean seeds having undergone microwave irradiation (500 W, for 30 s) prior to sprouting, the secondary metabolism has been activated of the synthesis of polyphenolic compounds. This, in turn, resulted in increased activity of superoxide dismutase, proportional to the increased synthesis of phenolic compounds. The author noted a 7-fold increase in antioxidant activity of such sprouts 6 days after initiating the process of sprouting compared to the seeds only soaked. The author also claims that microwaves induce heat stress, which in turn activates the phenylpropanoid metabolism pathway responsible for enhancing synthesis of phenolic compounds. Sales and Resurreccion [26] reported a particularly advantageous, ranging from 300 up to 2000%, increase in caffeic, coumaric, and ferulic acids in peanuts due to a combination of UV-C radiation and ultrasound. It should however be emphasized that the level of the mentioned compounds was not reflected in an increase in antioxidant activity measured by the ORAC test and total polyphenol content determined by the standard Folin’s method. On the other hand, Alothman et al. [27], who applied UV light to irradiate slices of banana, guava and pineapple for 10, 20 and 30 min, found increases in polyphenol content and total flavonols, in general, of 65% and of 40% in the first two; in the pineapple there was a 10% decrease in the contents of both groups of substances. The effects of heat stress can also be observed during the exposure to the electromagnetic field.

The vibrations of the electromagnetic field increase the temperature inside a cell, activating its antioxidant defense mechanisms. According to Shabrangi and Majd [28], various biological effects can be achieved by changing the power of the electromagnetic fields and duration of the exposure. Low frequency fields act more destructively on mitochondria through disturbances in calcium channels, thereby reducing the synthesis of antioxidants [29]. Effects of the exposure to electromagnetic fields depend also on the dry matter content. Nabizadeh et al. [30] reported that both 15 and 30-min exposure of dry and soaked pumpkin seeds to the electromagnetic field (2 mT) led to differentiation of the analyzed parameters. Both 15- and 30-min action of this field resulted in a fall in protein content as well as in the activity of peroxidase and dismutase in both types of pumpkin seeds. In the case of catalase, an increase in its activity was found only in the dry seeds treated with electromagnetic field for 15 min. A distinct diversity effect of magnetic field (2 mT) was recorded with regard to peroxidase. The soaked seeds were found to have higher activity of this enzyme, while in dried ones there was a drop below the activity recorded in a control sample. This was a pilot study and further research is needed. Long lasting activity of ultrasounds may produce excessive stress in cells of the plant, which will contribute to a decrease in the contents of polyphenolic compounds. Further detailed research is required to define optimum activity.

It should be emphasized that a short operation time along with the progress observed in technological development of machinery allows the use of microwave and ultrasonic technologies in industry to produce food containing enlarged amounts of biologically active compounds. The results obtained in this work indicate a particularly beneficial effect of such abiotic stress factors like ultrasound and microwave radiation on an increase in the content of polyphenolic compounds in chokeberry fruit. For three times of ultrasonication, which have been applied for eight anthocyanin compounds (in total 24 anthocyanin analysis), significant increases were recorded in fifteen cases. In the case of microwave radiation, a significant increase in their contents was found in seven out of the twenty-four cases examined. As for phenolic acids, an increase was significant for ten of the eighteen substances analyzed (three times of operation and six substances) which were exposed to ultrasound and for the eight subjected to microwave radiation. In turn, with regard to flavan-3-ols when ultrasonication was applied, an increase in their concentration was significant in twenty-four out of the thirty-three cases which were examined (three times of operation and eleven substances) and in thirty-two cases after microwave irradiation. In contrast, the application of ultrasonication and microwave radiation resulted in a significant increase of flavan-3-ols, respectively in seven and eight out of fifteen cases (three operating times and five identified substances). Of the abiotic stress factors employed, the statistically significant reduction in the content of all polyphenolic compounds determined in chokeberry fruits has been found only in the case of the exposure to 5-MFL (electromagnetic field) agent.

Based on the literature overview it should be noted that there are few works in the literature dealing with the effects of abiotic stress factors on changes occurring in the content of polyphenolic compounds in fruit. Therefore, further studies should be undertaken to broaden a current state of knowledge about the stimulating effect of various abiotic stress agents on the increased content of polyphenolic compounds in fruit.

## 4. Materials and Methods

### 4.1. Reagents and Standards

Methanol and formic acid were from Sigma-Aldrich (Steinheim, Germany). Acetonitrile was from Merck (Darmstadt, Germany). (−)-Epicatechin, (+)-catechin, procyanidin B2 chlorogenic acid, neochlorogenic acid, cryptochlorogenic acid, di-caffeic quinic acid, *p*-coumaric acid, quercetin-3-*O*-galactoside, quercetin-3-*O*-glucoside, quercetin-3-*O*-rutinoside, isorhamnetion-3-*O*-rutinoside, isorhamnetion-3-*O*-glucoside, eriodictyol 7-*O*-glucuronide, cyanidin-3-*O*-galactoside, cyanidin-3-*O*-glucoside, cyanidin-3-*O*-arabinoside and cyanidin-3-*O*-xyloside were purchased from Extrasynthese (Lyon, France).

### 4.2. The Experimental Material

The experimental material consisted of chokeberry fruits of the cultivar Galicyjanka harvested at the technological stage of maturity in August 2015 on a plantation around Rzeszów, Niechobrz Poland (N 49°58′52.9788′′, E 21°50′40.7328′′). Chokeberry (*Aronia melanocarpa* Elliot) fruits cv. Galicjanka were collected at the optimum ripening stage recommended for consumption. After harvest, chokeberry fruits were washed. The entire batch was then divided into fifteen 500 g portions, of which one, a control sample (coded 1), was analyzed for the content of selected phenolic compounds without exposure to the stress factors. The remainder were exposed to the following abiotic stress factors: UV-C radiation using a NBV 30N lamp (UltraViol, Zgierz, Poland) with radiation intensity of 2.3 W/m^2^; an electromagnetic field (150 μT, 100 Hz); microwaves (100 W and 180 W); and ultrasonication (800 W, 40 Hz). For these stress agents, various operating times have been applied. The sample codes along with the corresponding stress factor and its operating time are given below.

1. CP—for a control sample;
2. UV—for UV-C irradiation by for 20 min;
3. UV—for UV-C irradiation for 40 min;
4. UV—for UV-C irradiation for 60 min;
5. MFL—for an electromagnetic field (150 μT, 100 Hz, 30 min);
6. MFL—for an electromagnetic field (150 μT, 100 Hz, 60 min);
7. MFH—for an electromagnetic field (40 mT, 50 Hz, 0.5 min);
8. MFH—for an electromagnetic field (40 mT, 50 Hz, 2 min);
9. MFH—for an electromagnetic field (40 mT, 50 Hz, 5 min);
10. MW—for microwaves (100 W, 1 min);
11. MW—for microwaves (100 W, 2 min);
12. MW—for microwaves (180 W, 1 min);
13. US—for ultrasonication (800 W, 40 Hz, 2 min);
14. US—for ultrasonication (800 W, 40 Hz, 4 min);
15. US—for ultrasonication (800 W, 40 Hz, 8 min).

After each treatment, fruits was kept at room temperature for 30 min, then frozen in liquid nitrogen and subjected to 24 h freeze-drying in an Alpha 1–4 LSC freeze dryer (Christ, Germany). After drying they were crushed using an IKA 11A laboratory mill (BIOSAN, Vilnius, Lithuania), hermetically sealed in ziploc bags and stored at −70 °C in a Frilabo freezer (Lyon, France) until extraction, but not for longer than 30 days.

### 4.3. Extraction

Polyphenolic compounds were isolated by means of extraction supported by ultrasounds. First, 0.5 g of the finely ground material was placed in a Falcon centrifuge tube (50 mL), to which 50% aqueous methanol containing formic acid (1%) was poured. The extraction was performed twice by incubation for 20 min under sonication (Sonic 6D, Polsonic, Warsaw, Poland) and with occasional shaking. Next, the slurry was centrifuged at 19,000 *g* for 10 min, and the supernatant was filtered through a Hydrophilic PTFE 0.20 μm membrane (Millex Samplicity Filter, Merck, Darmstadt, Germany) and used for analysis. The content of polyphenols in individual extracts was determined by means of the UPLC-PDA-MS/MS method [31]. All extractions were carried out in triplicate.

### 4.4. Identification and Quantification of Polyphenols by the UPLC-PDA-MS Method

**Figure 5:**
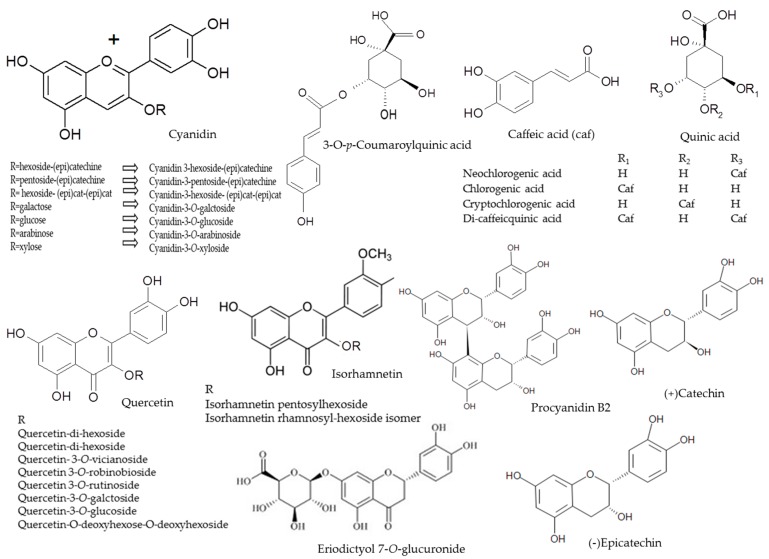
Chemical structures of polyphenolic compounds present in chokeberries.

**Figure 6:**
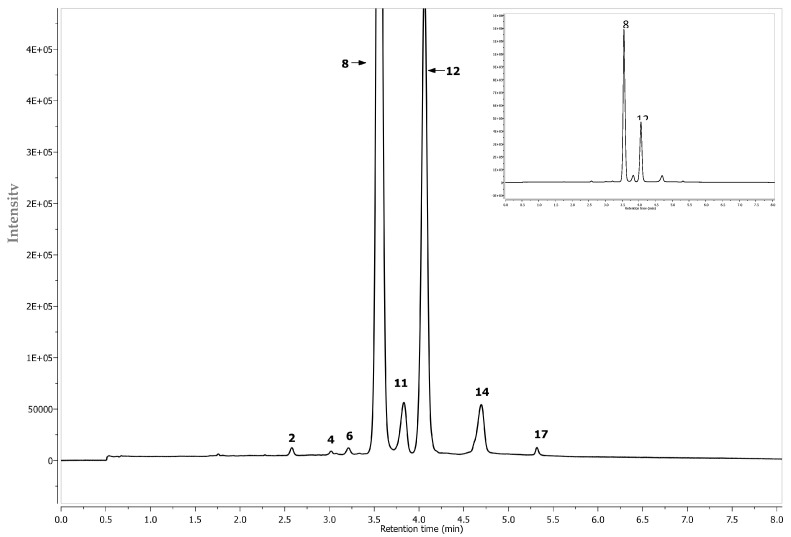
Segment from 0.0 to 8.0 min of LC-DAD chromatogram at 520 nm of chokeberry extracts. Peak number identities are displayed in Table 2.

**Figure 7:**
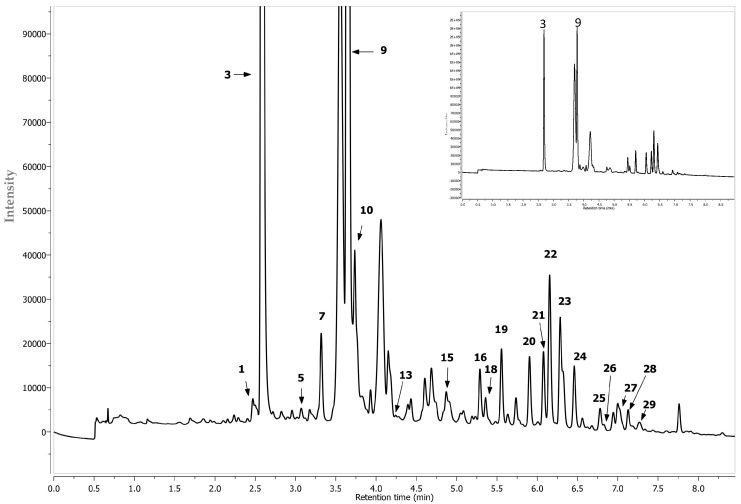
Segment from 0.0 to 9.0 min of LC-DAD chromatogram at 280 nm of chokeberry extracts. Peak number identities are displayed in Table 2.

Phenolic compounds were determined by an Aquity Ultra-Performance Liquid Chromatograph, equipped with a Binary Solvent Manager (BSM), a Sample Manager (SM) coupled with a PDA detector and a Quadrupole–Time Of Flight (Q–TOF) tandem mass detector (Waters, Manchester, UK). Separations were carried out on a 2.1 × 100 mm UPLC BEH C18 column containing 1.7 µm particles (Waters, Manchester, UK). Isocratic-gradient elution was chosen as the elution mode, in which were used: 2% aqueous formic acid (A) and acetonitrile (B), at the mobile phase velocity of 0.45 mL/min. Elution was initiated at 99% A for one minute, then a linear gradient was applied to 75% B in 12 min. The column temperature was 30 °C and the volume of injections 5 µL. Operating parameters of the mass detector were as follows: capillary voltage of 2.5 kV and the sampling cone voltage of 30 V. Temperatures of the ion source and desolvation were 130 °C and 350 °C, respectively. Nitrogen, at a flow rate of 300 L/h, was used as carrier gas. Analyses were carried out in a full scan mode within the range 100–1500 *m*/*z*, upon the tolerance of 0.001 Da and resolution of 5000. The internal reference standards, leucine and enkephalin, were introduced continuously through lockspray reference channel. The chromatograms were analyzed employing the base peak (BPI) calibrated to 12,400 cps (100%). Data were collected and analyzed using MassLynx v4.1 software (Waters). Anthocyanins were analyzed in the positive-ion mode, while the remaining polyphenols in the negative-ion mode. Their identification was done by comparing spectra of maximum UV-radiation absorption, molecular weight determined as the mass/charge ratio, retention times, as well as fragmentation spectra, with the available literature data (Table 2 and Figure 5) [32,33,34]. Decay spectra were obtained as a result of collision-induced dissociation (CID) in the tandem mode. Collision energy was selected individually for each substance analyzed. The characteristic UV-spectra were collected at the following wavelengths: λ = 520 nm (Figure 6), anthocyanins; λ = 320 (Figure 7), phenolic acids; λ = 360 (Figure 7), flavonols; and λ = 280 (Figure 7), flavan-3-ols. The quantification of phenolic compounds was performed by external calibration curves, using reference compounds selected based on the principle of structure-related target analyte/standard (chemical structure or functional group). The calibration curve for *p*-coumaric acid was used to quantify 3-*O*-*p*-coumaroylquinic acid. Chlorogenic, cryptochlorogenic, neochlorogenic and di-caffeic quinic acids were quantified with their own standards. (+)Catechin, (−)Epicatechin and procyanidin B2 were quantified with its own standard. Eriodictyol 7-*O*-glucuronide was quantified with their own standards. The calibration curves of quercetin-3-*O*-rutinoside, 3-*O*-glucoside and 3-*O*-galactoside were used to quantify quercetin derivatives. The calibration curves of cyanidin-3-*O*-glucoside, -3-*O*-galactoside, -3-*O*-arabinoside, -3-*O*-xyloside, -3-hexosode(epi)catechine, -3-pentoside-(epi)catechine and -3-hexoside-(epi)cat-(epi)cat were used to quantify cyanidin derivatives. Cyanidin-3-*O*-glucoside, -3-*O*-galactoside, -3-*O*-arabinoside, -3-*O*-xyloside were quantified with their own standards. For isorhamnetin quantification, isorhamnetin 3-*O*-rutinoside and 3-*O*-glucoside were used. All determinations were done in triplicate (*n* = 3). The standards have been prepared in concentrations ranging between 0.05–5 mg/mL. The correlation coefficient was *R*^2^ ≤ 0.9998. Results were expressed in mg per g of dm.

### 4.5. Statistical Analysis

The results were statistically evaluated using an analysis of medium significance and cluster analysis, the procedures available in the Statistica version 10.0 (StatSoft, Tulosa, OK, USA) software package for statistical data processing. Medium significance was evaluated by the test of independent pairs (Student’s *t*-test) at a significance level of α = 0.05. Cluster analysis allowed the display of inter-factors links related to the effect of abiotic stress factors on the diversity in polyphenols content in black chokeberry.

## 5. Conclusions

The use of abiotic stress factors, like UV-C radiation, microwaves, and ultrasound field, in the vast majority of the applied factors contributed to an increase in the content of most of the analyzed polyphenolic compounds in black chokeberry. In contrast, the application of the electromagnetic field led to decreases of these compounds. The results obtained allow us to indicate the possible commercial use of microwave radiation and ultrasound to produce, based on chokeberry fruit, functional food with increased content of anthocyanins, phenolic acids, flavonols and flavan-3-ols.

## Figures and Tables

**Table 1:** The influence of abiotic stress factors (UV-C electromagnetic field of the microwave field, ultrasound) on the content of individual polyphenolic compounds in chokeberry fruits, mg/100 g dm (*n* = 3).

	Abiotic Stress Factors														
	CP	UV			MFL		MFH			MW			US		
	Control	20	40	60	30	60	0.5	2	5	1	2	0.5	2	4	8
*Anthocyanins*															
Cyanidin-3-hexoside-(epi)catechin	11.70 **	13.90 *	12.90 *	16.10 *	9.10 *	12.50	12.60 *	12.00	5.99 *	15.40 *	14.10 *	12.80 *	12.30	16.90 *	15.70 *
Cyanidin-3-pentoside-(epi)catechin	8.78 **	8.08	9.19	10.30 *	6.03 *	8.33	9.82	8.84	4.09 *	10.30	9.81	8.96	8.13	11.30 *	10.20 *
Cyanidin-3-hexoside- (epi)cat-(epi)cat	14.90 **	15.40	13.80	18.20 *	9.95 *	13.50	16.30	14.10	4.30 *	10.30 *	18.20 *	15.80	13.00 *	19.40 *	17.50 *
Cyanidin-3-*O*-galactoside	3367 **	3607	3637	3394	2322 *	2945 *	3405	3311	2608 *	3717	3678	3266	3050	3797 *	4033 *
Cyanidin-3-*O*-glucoside	149 **	164	158	155	108 *	152	164	159.5	119 *	164	173 *	155	133	170 *	188 *
Cyanidin-3-*O*-arabinoside	1454 **	1579	1584	1501	1002 *	1309	1516	1462	1119 *	1596	1617	1391	1314	1635 *	1826 *
Cyanidin-3-*O*-xyloside	165 **	171	181	164	127 *	168	176	174	129 *	174	185	174	145 *	186 *	219 *
Cyanidin	2.68 **	4.32 *	3.48 *	2.58	1.89 *	1.95	4.91 *	4.69 *	2.54	4.71 *	2.92	5.13 *	3.32 *	2.00 *	6.25 *
*Phenolic acids*															
Neochlorogenic acid	1250 **	1276	1087 *	1242	858 *	1372	1396	1367	997 *	1225	1380	1180	1167	1418 *	1340
3-*O*-p-coumaroylquinic acid	10.7 **	11.3	9.71	11.2	7.63 *	12.7 *	12.6 *	12.3 *	8.7 *	11.2	13.0 *	10.9	10.7	12.4 *	12.1 *
Chlorogenic acid	740 **	862 *	806	831 *	592 *	913 *	884	886 *	729	847 *	970 *	868 *	793	976 *	894 *
Cryptochlorogenic acid	25.5 **	27.9	36.4 *	29.3 *	19.2 *	33.6 *	31.0 *	29.9 *	27.1	28.6	33.2 *	27.0	26.3	31.9 *	31.4 *
Di-caffeic quinic acid I	3.76 **	3.83	3.37	3.92	2.48 *	3.77	4.14	4.16	3.17 *	3.73	4.17	3.88	3.61	4.47 *	3.99
Di-caffeic quinic acid II	0.86 **	0.28 *	0.56 *	0.51 *	0.54 *	0.94	0.96	1.05 *	0.78	1.21 *	0.85	0.72 *	0.73 *	1.09 *	1.5 *
*Flavonols*															
Quercetin-dihexoside	10.90 **	13.40 *	11.80	14.10 *	9.24 *	16.50 *	15.50 *	14.80 *	11.60	14.90 *	16.10 *	15.70 *	12.80 *	17.30 *	14.50 *
Quercetin-dihexoside	5.63 **	6.71 *	6.15	6.58 *	4.37 *	8.16 *	7.79 *	6.92 *	5.64	7.23 *	8.01 *	7.00 *	6.33 *	8.25 *	7.34 *
Quercetin-3-*O*-vicianoside	14.20 **	16.20 *	14.30	18.60 *	11.90 *	22.00 *	21.20 *	19.30 *	15.70	19.60 *	21.30 *	21.10 *	17.20 *	22.10 *	18.80 *
Quercetin-3-*O*-robinobioside	22.50 **	24.90	24.30	26.20 *	16.10 *	25.20	26.30 *	23.80	18.70 *	27.40 *	28.20 *	23.40 *	20.90	28.50 *	27.60 *
Quercetin-3-*O*-rutinoside	31.70 **	36.20 *	33.30	38.60 *	24.30 *	40.10 *	40.60 *	36.00 *	28.90	39.80 *	42.00 *	36.40 *	32.60	44.70 *	39.40 *
Quercetin-3-*O*-galctoside	39.90 **	48.50 *	44.20	51.60 *	33.00 *	52.10 *	56.00 *	47.00 *	39.40	57.90 *	54.90 *	46.10 *	45.50 *	60.20 *	54.60 *
Quercetin-3-*O*-glucoside	33.20 **	39.10 *	36.40	40.30 *	25.80 *	40.90 *	43.00 *	36.50	31.40	44.00 *	44.30 *	35.40 *	35.50	46.20 *	43.80 *
Isorhamnetin pentosylhexoside	1.62 **	1.57	1.59	1.74	1.17 *	1.39 *	1.77	1.72	1.27 *	1.90 *	2.28 *	1.73	1.54	2.00 *	2.12 *
Quercetin-*O*-deoxyhexose-*O*-deoxyhexoside	6.91 **	8.29 *	8.33 *	8.41 *	5.37 *	8.84 *	8.94 *	8.72 *	6.96	9.43 *	9.78 *	8.42 *	7.23	9.77 *	9.32 *
Isorhamnetin rhamnosylhexosideisomer	1.13 **	1.36 *	1.39 *	1.41 *	0.96 *	1.77 *	1.65 *	1.64 *	1.07	1.35 *	1.84 *	1.41 *	1.23	2.12 *	1.37 *
Isorhamnetin rhamnosylhexosideisomer	1.04 **	1.05	1.86 *	1.06	1.00	1.02	1.00	1.96 *	1.69 *	1.37 *	1.34 *	1.56 *	1.23	1.42 *	1.24 *
*Flavan-3-ols*															
Procyanidin B2	66.6 **	55.6 *	66.4	77.9 *	46.1 *	86.0 *	80.1 *	78.3 *	48.8	70.3	77.7 *	68.8	65.1	92.0 *	81.6 *
(+)Catechin	71.4 **	79.9	36.5 *	80.7 *	56.3 *	101.8 *	86.1 *	79.7	35.9 *	82.0 *	93.6 *	78.5	70.3	93.9 *	88.6 *
Procyanidin B2	25.7 **	23.5	21.2 *	23.8	17.4 *	30.7 *	24.8	32.2 *	11.7 *	22.8 *	26.8	20.1 *	24.6	29.0 *	27.3
(−)Epicatechin	62.0 **	63.6	57.6	64.3	41.0 *	75.3 *	69.1	70.1 *	40.2 *	65.6	72.9 *	58.5	59.3	79.7 *	67.9
*Flawanones*															
Eriodictyol 7-*O*-glucuronide	58.0 **	67.30 *	58.60	63.00	42.60 *	65.20 *	41.20	64.10	54.70	67.00 *	71.60 *	58.40	60.80	74.90 *	73.30
ΣPC	7125	7659,00	7453	7287	5009	6859	7541	7200	5701	7723	8003	7034	6602	8182	8500

* Statistically significant differences compared to the control at significance level α = 0.05.

**Table 2:** Identification of phenolic compounds in black chokeberry (*Aronia melanocarpa* L.) fruits by ultra-performance liquid chromatography and photodiode detector-quadrupole/time-of-flight mass spectrometry (UPLC-PDA-MS/MS).

Peak	Assigned Identity	RT	[M − H]^+^	Fragment Ions	Absorbance Maxima
No.		(min)	(*m*/*z*)	(*m*/*z*)	(nm)
1	Procyanidin B2 ^2^	2.46	577	425, 289	280
2	Cyanidin-3-hexoside-(epi)catechin ^2^	2.55	737 ^+^	575, 423, 287	242, 520
3	Neochlorogenic acid ^1^	2.56	353	191 179	322
4	Cyanidin-3-pentoside-(epi)catechin ^2^	2.99	707 ^+^	557, 329, 287	283, 524
5	(+) Catechin ^1^	3.04	289	-	283
6	Cyanidin-3-hexoside- (epi)cat-(epi)cat ^2^	3.17	1025 ^+^	575, 409, 287	283, 519
7	3-*O*-*p*-Coumaroylquinic acid ^2^	3.3	337	191	310
8	Cyanidin-3-*O*-galctoside ^1^	3.51	449 ^+^	287	279, 514
9	Chlorogenic acid ^1^	3.62	353	191, 179	322
10	Cryptochlorogenic acid ^1^	3.71	353	191, 179	318
11	Cyanidin-3-*O*-glucoside ^1^	3.81	449 ^+^	287	279, 514
12	Cyanidin-3-*O*-arabinoside ^1^	4.03	419 ^+^	287	279, 514
13	Procyanidin B2 ^1^	4.21	577	425, 289	280
14	(−)Epicatechin ^1^	4.89	289	-	277
15	Cyanidin-3-*O*-xyloside ^1^	4.68	419 ^+^	287	279, 514
16	Quercetin-di-hexoside ^2^	5.26	625	445, 301	255, 353
17	Cyanidin ^2^	5.29	287 ^+^	287	280, 514
18	Quercetin-di-hexoside ^2^	5.33	625	445,301	263, 355
19	Quercetin-3-*O*-vicianoside (or Quercetin-6-*O*-arabinopyranosyl-d-glucopyranose (3-*O*-vicianoside) ^2^	5.52	595	432, 301	255, 353
20	Quercetin-3-*O*-robinobioside (or Quercetin 6-*O*-(6-deoxy-α-L-manno pyranosyl)-β-d-galactopyranose (3-*O*-robinobioside)) ^2^	5.87	609	463, 301	231, 323
21	Quercetin-3-*O*-rutinoside (or Quercetin 6-*O*-alpha-l-rhamnopyranosyl-d-glucopyranose (3-*O*-rutinoside)) ^1^	6.04	609	463, 301	255, 352
22	Quercetin-3-*O*-galctoside ^1^	6.12	463	301	255, 352
23	Quercetin-3-*O*-glucoside ^1^	6.25	463	301	255, 352
24	Eriodictyol 7-*O*-glucuronide	6.33	280	463	287
25	Isorhamnetin pentosylhexoside ^2^	6.59	609	477, 315	264, 352
26	Quercetin-*O*-deoxyhexose-*O*-deoxyhexoside ^2^	6.75	593	433, 301	256, 352
27	Isorhamnetin rhamnosyl-hexoside isomer ^2^	6.79	623	463, 315	264, 345
28	Di-caffeic quinic acid ^1^	7.10	515	353, 191	312
29	Isorhamnetin rhamnosyl-hexoside isomer ^2^	7.15	623	421, 315	264, 345
30	Di-caffeic quinic acid ^1^	7.25	515	353, 179	308

^1^ Identification confirmed by commercial standards. ^2^ Identification by comparison of MS data with literature and their identification is tentative.

## References

[1] Šnebergrová J., Čížková H., Neradová E., Kapci B., Rajchl A., Voldřich M. (2014). Variability of Characteristic Components of Aronia. Czech J. Food Sci..

[2] Kulling S.E., Rawel H.M. (2008). Chokeberry (*Aronia melanocarpa*)—A Review on the Characteristic Components and Potential Health Effects. Planta Med..

[3] Oszmiański J., Wojdyło A. (2005). Aronia melanocarpa phenolics and their antioxidant activity. Eur. Food Res. Technol..

[4] Park S., Kim J.I., Lee I., Lee S., Hwang M.W., Bae J.Y., Heo J., Kim D., Han S.Z., Park M.S. (2013). Aronia melanocarpa and its components demonstrate antiviral activity against influenza viruses. Biochem. Biophys. Res. Commun..

[5] Kokotkiewicz A., Jaremicz Z., Luczkiewicz M. (2010). Aronia Plants: A Review of Traditional Use. Biological Activities. And Perspectives for Modern Medicine. J. Med. Food.

[6] Ho G.T.T., Bräunlich M., Austarheim I., Wangensteen H., Malterud K.E., Slimestad R., Barsett H. (2014). Immunomodulating Activity of Aronia melanocarpa Polyphenols. Int. J. Mol. Sci..

[7] Fang J. (2015). Classification of fruits based on anthocyanin types and relevance to their health effects. Nutrition.

[8] Quinlan C.L., Perevoshchikova I.V., Hey-Mogensen M., Orr A.L., Brand M.D. (2013). Sites of reactive oxygen species generation by mitochondria oxidizing different substrates. Redox Biol..

[9] Yao L.H., Jiang Y.M., Shi J., Tomas-Barber F.A., Datta N., Singanusong R., Chen S.S. (2004). Flavonoids in Food and Their Health Benefits. Plant Foods Hum. Nutr..

[10] Lachowicz S., Oszmiański J., Pluta S. (2017). The composition of bioactive compounds and antioxidant activity of Saskatoon berry (*Amelanchier alnifolia* Nutt.) genotypes grown in central Poland. Food Chem..

[11] Bochkov D.V., Sysolyatin S.V., Kalashnikov A.I., Surmacheva I.A. (2012). Shikimic acid: Review of its analytical, isolation, and purification techniques from plant and microbial sources. J. Chem. Biol..

[12] Kováčik J., Klejdu B., Bačkor M., Repčak M. (2007). Phenylalanine ammonia-lyase activity and phenolic compounds accumulation in nitrogen-deficient Matricaria chamomilla leaf rosettes. Plant Sci..

[13] Gill S.S., Tuteja N. (2010). Reactive oxygen species and antioxidant machinery in abiotic stress tolerance in crop plants. Plant Physiol. Biochem..

[14] Ke D., Saltveit M.E. (1988). Plant Hormone Interaction and Phenolic Metabolism in the Regulation of Russet Spotting in Iceberg Lettuce. Plant Physiol..

[15] Hasanuzzaman M., Hossain M.A., Teixeira da Silva J.A., Fujita M. (2012). Plant Response and Tolerance to Abiotic Oxidative Stress: Antioxidant Defense Is a Key Factor. Crop Stress Manag. Perspect. Strateg..

[16] Yu J., Engeseth N.J., Feng H. (2016). High Intensity Ultrasound as an Abiotic Elicitor—Effects on Antioxidant Capacity and Overall Quality of Romaine Lettuce. Food Bioprocess Technol..

[17] Mittler R. (2002). Oxidative stress. Antioxidants and stress tolerance. Trends Plant Sci..

[18] Mirouze M., Paszkowski J. (2011). Epigenetic contribution to stress adaptation in plants. Curr. Opin. Plant Biol..

[19] Kosová K., Vítámvása P., Prášila I.T., Renautb J. (2011). Plant proteome changes under abiotic stress—Contribution of proteomics studies to understanding plant stress response. J. Proteom..

[20] Slavin J.L., Lloyd B. (2012). Health Benefits of Fruits and Vegetables. Adv. Nutr..

[21] Liu C., Cai L.Y., Lu X.Y., Han X.X., Ying T.J. (2012). Effect of Postharvest UV-C Irradiation on Phenolic Compound Content and Antioxidant Activity of Tomato Fruit During Storage. J. Integr. Agric..

[22] Kwiatkowska B., Bennett J., Akunna J., Walker G.M., Bremner D.H. (2011). Stimulation of bioprocesses by ultrasound. Biotechnol. Adv..

[23] Wu J., Lin L. (2002). Ultrasound-Induced Stress Responses of Panax ginseng Cells: Enzymatic Browning and Phenolics Production. Biotechnol. Prog..

[24] Santos J.G., Fernandes F.A.N., Oliveira L.S., Miranda M.R.A. (2015). Influence of Ultrasound on Fresh-Cut Mango Quality through evaluation of enzymatic and oxidative metabolism. Food Bioprocess Technol..

[25] Randhir R., Shetty K. (2004). Microwave-induced stimulation of l-DOPA, phenolics and antioxidant activity in fava bean (*Vicia faba*) for Parkinson’s diet. Process Biochem..

[26] Sales J.M., Resurreccion A.V.A. (2010). Phenolic profile, antioxidants and sensory acceptance of bioactive-enhanced peanuts using ultrasound and UV. Food Chem..

[27] Alothman M., Bhat R., Karim A.A. (2009). UV radiation-induced changes of antioxidant capacity of fresh-cut tropical fruits. Innov. Food Sci. Emerg..

[28] Shabrangi A., Majd A. (2009). Efect of Magnetic Fields on Growth and Antioxidant Systems in Agricultural Plants. PIERS Proc..

[29] Belyavskaya N.A. (2004). Biological effects due to weak magnetic field on plants. Adv. Space. Res..

[30] Nabizadeh S., Majd A., Arbabiyan S., Mirzai M., Sharifnia F. (2014). Assessment of the Effect of Electromagnetic Fields on Biochemical and Antioxidant Parameter Changes of Cucurbita maxima Duchesne. Adv. Environ. Biol..

[31] Lachowicz S., Oszmiański J., Seliga Ł., Pluta S. (2017). Phytochemical Composition and Antioxidant Capacity of Seven Saskatoon Berry (*Amelanchier alnifolia* Nutt.) Genotypes Grown in Poland. Molecules.

[32] Lee J.E., Kim G.S., Park S., Kim Y.H., Kim M.B., Lee W.S., Jeong S.W., Lee S.J., Sung J.J., Shin S. (2014). ChDetermination of chokeberry (*Aronia melanocarpa*) polyphenol components using liquid chromatography–tandem mass spectrometry: Overall contribution to antioxidant activity. Food Chem..

[33] Mikulic-Petkovsek M., Slatnar A., Stampar F., Veberic R. (2012). HPLC–MS identification and quantification of flavonol glycosides in 28 wild and cultivated berry species. Food Chem..

[34] Oszmiański J., Lachowicz S. (2016). Effect of the production of dried fruits and juice from chokeberry (*Aronia melanocarpa* L.) on the content and antioxidative activity of bioactive compounds. Molecules.

